# Influence of zinc-modified niobium nanoparticles incorporation in resinous monomers on mechanical properties

**DOI:** 10.1590/0103-644020266930

**Published:** 2026-08-07

**Authors:** Larissa Luri Almeida Amorim Ikejiri, Marilia Mattar de Amoêdo Campos Velo, Nair Cristina Margarido Brondino, Carlos Alberto Spironelli Ramos, Ana Paula de Melo Alves Guedes, Tatiana Rita de Lima Nascimento, Rafael Francisco Lia Mondelli

**Affiliations:** 1 Department of Restorative Dentistry, Endodontics and Dental Materials, Bauru School of Dentistry, Bauru, São Paulo, Brazil.; 2 Department of Restorative Dentistry, Araraquara School of Dentistry, Araraquara, São Paulo, Brazil; 3 Department of Mathematics, São Paulo State University, Bauru, São Paulo, Brazil; 4 Department of Endodontics, State University of Londrina, Londrina, Paraná, Brazil; 5 Departamento de Chemistry, Federal University of Paraiba, João Pessoal, Paraíba, Brazil

**Keywords:** nanoparticles, Niobium, composite resin, zinc

## Abstract

This *in vitro* study evaluated the influence of the incorporation of different concentrations of zinc-modified niobium nanoparticles (Nb/Zn) on the mechanical properties of a flowable resin composite (OptiFlow II, LC/Pac-Dent) by surface hardness (SH), depth of cure (DoC), flexural strength (FS), and modulus of elasticity (*E*). The morphology and composition of Nb/Zn nanoparticles were evaluated by Scanning Electron Microscopy and energy-dispersive X-ray spectroscopy (EDX). The Nb/Zn nanoparticles were incorporated at 3 different concentrations (0.3%; 0.6% e 0.9%), resulting in four groups: OptiFlowII, OptiFlow II+0.3%Nb/Zn, OptiFlow II+0.6%Nb/Zn, and OptiFlow II+0.9%Nb/Zn. SH was measured at the top and bottom of the specimens (n=6/group). FS and *E* were conducted using a Universal Testing Machine (Instron, 50N) (n=14/group). For SH, DoC, and FS, ANOVA followed by Tukey's test was used for statistical analyses, and for *E*, pairwise comparisons were conducted using a sandwich covariance matrix (p<0.05). The results showed that OptiFlow II+0.6%Nb/Zn exhibited the lowest top-SH values in all comparisons (p=0.003). Regarding DoC, the data indicated a significant difference between the groups with 0.3% and 0.6% Nb/Zn (p=0.04). For *E*, OptiFlow II+0.3%Nb/Zn presented higher values (*E*=3.48±0.15)^a^ compared to OptiFlow II+0.6%Nb/Zn (*E*=3.3±0.18; p=0.02)^b^, OptiFlowII presented higher values (*E*=3.56±0.27)^a^ than OptiFlow II+0.6%Nb/Zn (p=0.01) and OptiFlow II+0.9%Nb/Zn (*E*=3.3±0.27; p=0.02)^b^. Thus, the incorporation of Nb/Zn nanoparticles at different concentrations into the OptiFlow II affects its mechanical properties, without altering the depth of cure, but reducing flexural strength. The group with the lowest nanoparticle concentration showed *E* similar to that of the control group and higher than that of the higher-concentration groups. Overall, the incorporation of Nb/Zn nanoparticles influenced the mechanical behavior of the flowable resin composite. Lower concentrations performed similarly to the control, while higher levels impaired properties, highlighting the need for optimized nanoparticle loading.

## Resumo

Este estudo in vitro avaliou a influência da incorporação de diferentes concentrações de nanopartículas de nióbio modificadas com zinco (Nb/Zn) nas propriedades mecânicas de uma resina composta fluida (OptiFlow II, LC/Pac-Dent) por meio da dureza superficial (DS), profundidade de cura (PC), resistência à flexão (RF) e módulo de elasticidade (E). A morfologia e composição das nanopartículas Nb/Zn foram avaliadas por microscopia eletrônica de varredura (MEV) e espectroscopia de energia por dispersão de raios X (EDX). As nanopartículas Nb/Zn foram incorporadas em três concentrações diferentes (0,3%; 0,6% e 0,9%), resultando em quatro grupos: OptiFlow II; OptiFlow II+0,3%Nb/Zn; OptiFlow II+0,6%Nb/Zn; e OptiFlow II+0,9%Nb/Zn. A dureza superficial foi medida no topo e na base dos espécimes (n=6/grupo). A resistência à flexão e o módulo de elasticidade foram avaliados utilizando uma máquina universal (Instron, 50N) (n=14/grupo). Para DS, PF e RF, foram utilizadas ANOVA seguida do teste de Tukey para análise estatística, e para E, comparações pareadas foram realizadas usando matriz de covariância sandwich (p<0,05). Os resultados mostraram que o grupo OptiFlow II+0,6%Nb/Zn apresentou os menores valores de dureza superficial no topo em todas as comparações (p=0,003). Em relação à profundidade de cura, os dados indicaram diferença significativa entre os grupos com 0,3% e 0,6% Nb/Zn (p=0,04). Para E, o grupo OptiFlow II+0,3%Nb/Zn apresentou valores maiores (E=3,48±0,15)a em comparação ao OptiFlow II+0,6%Nb/Zn (E=3,3±0,18; p=0,02)b, e o grupo OptiFlow II apresentou valores maiores (E=3,56±0,27)a que o OptiFlow II+0,6%Nb/Zn (p=0,01) e OptiFlow II+0,9%Nb/Zn (E=3,3±0,27; p=0,02)b. Assim, a incorporação de nanopartículas de Nb/Zn em diferentes concentrações na resina OptiFlow II afeta suas propriedades mecânicas, sem alterar a profundidade de cura, mas reduzindo a resistência à flexão. O grupo com menor concentração de nanopartículas apresentou módulo de elasticidade semelhante ao grupo controle e maior em comparação aos grupos com maiores concentrações.

**Figure:**
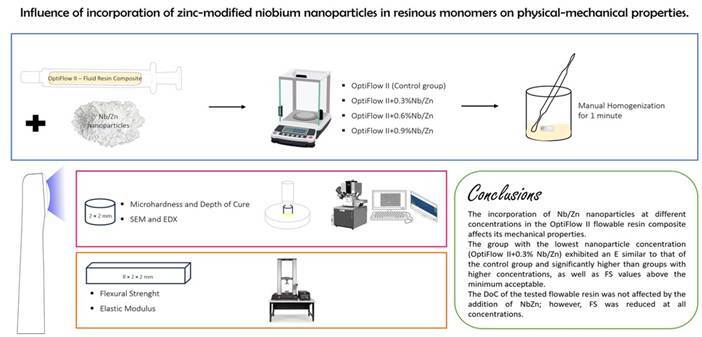

## Introduction

Resin composite is considered the material of choice for direct adhesive restorations in both anterior and posterior teeth due to its esthetic appearance, adhesive properties, and favorable biological response ^1^ ^,^ ^3^ ^,^ ^4^ ^,^ ^5^. Over the past decades, modifications in resin composite formulations have broadened their clinical utility. In 1996, formulations with lower viscosity were introduced, giving rise to materials commonly referred to as flowable resin composites ^4^ ^,^ ^6^. Compared with conventional resin composites, flowable resin composites contain a reduced concentration of inorganic fillers and a higher proportion of resin matrix, resulting in decreased viscosity, improved wettability, and superior adaptability to complex cavity geometries ^6^ ^,^ ^9^.

These rheological characteristics underline clinical preference for flowable resin composites in specific scenarios where intimate adaptation to the tooth structure is critical. Therefore, flowable resin composites are widely indicated for sealing enamel microdefects and pits, acting as liners in deep Class I and II preparations, repairing marginal discrepancies, and restoring non-carious cervical lesions, particularly in cases where a low viscosity material facilitates precise placement and reduces operator dependency ^6^ ^,^ ^7^ ^,^ ^8^ ^,^ ^9^ ^,^ ^25^. Their enhanced adaptability allows the material to penetrate irregularities and fine microfeatures that are difficult to access with more viscous conventional resin composites, making them especially suited to minimal-intervention approaches in modern adhesive dentistry.

Despite these clinical advantages, the inherent limitation associated with the lower filler content is a compromise in the mechanical integrity and long term performance of flowable resin composites. Several studies have demonstrated that reduced inorganic filler loading is associated with inferior structural behavior, greater polymerization shrinkage, and greater susceptibility to deformation under functional loading compared with conventional resin composites ^6^ ^,^ ^9^ ^,^ ^26^. Such limitations contribute to an elevated risk of marginal gaps, microleakage, and caries development adjacent to restorations, factors that remain among the primary causes of restoration failure in clinical practice ^6^ ^,^ ^9^ ^,^ ^27^. Thus, although flowable resin composites offer handling and placement advantages, their mechanical fragility and limited bioactivity limit their use as definitive restorative materials under occlusal load, underscoring the need for materials that maintain clinical adaptability without compromising durability.

To overcome the inherent mechanical limitations of flowable resin composites, several strategies have been explored to reinforce their structure. In this context, nanotechnology has emerged as a promising approach, enabling the incorporation of nanoscale fillers to improve the mechanical behavior and structural stability of resin-based materials ^2^ ^,^ ^6^ ^,^ ^12^ ^,^ ^15^ ^,^ ^16^ ^,^ ^17^ ^,^ ^18^ ^,^ ^19^ ^,^ ^20^ ^,^ ^21^. Among these, zinc (Zn) nanoparticles have attracted attention due to their compatibility with resin matrices and their potential to enhance material stiffness and resistance to deformation ^1^ ^,^ ^12^ ^,^ ^19^ ^,^ ^22^ ^,^ ^28^. Despite these advantages, Zn-based nanoparticles alone may present limitations when used as reinforcing agents in resin composites. Previous studies have reported that although ZnO nanoparticles may provide some mechanical reinforcement, excessive concentrations can promote particle agglomeration, compromising the composite's homogeneity and negatively affecting its mechanical performance ^19^ ^,^ ^22^ ^,^ ^28^. Therefore, the exploration of complementary nanostructured additives capable of further improving the physicochemical stability and mechanical behavior of resin composites remains an important research focus.

Niobium nanoparticles (Nb), a transition metal with favorable physicochemical properties, have emerged as promising additives in dental materials. Niobium-containing compounds have demonstrated high chemical stability, radiopacity, and favorable interaction with polymer matrices, contributing to improved mechanical behavior and structural durability of resin-based materials ^13^ ^,^ ^16^ ^,^ ^24^ ^,^ ^30^ ^,^ ^31^ ^,^32^)^. In addition, niobium-based nanostructures have shown potential to enhance filler-matrix interactions and reduce degradation in polymeric systems. 

In this context, the development of zinc-doped niobium (Nb/Zn) nanoparticles represents a rational strategy to combine the structural stability associated with niobium-based fillers with the functional properties of zinc. Beyond the well-known antibacterial potential of Zn, its incorporation into niobium nanostructures may also modify surface characteristics, improve particle dispersion within the resin matrix, and reduce nanoparticle agglomeration, thereby contributing to improved physicochemical stability and mechanical performance of resin composites. Despite the growing interest in these nanomaterials individually, the incorporation of zinc-doped niobium nanoparticles into flowable resin composites remains poorly explored. This represents a relevant knowledge gap, particularly considering the need to develop flowable restorative materials with improved mechanical performance and structural stability. Thus, the present study focused on evaluating the mechanical behavior of a flowable resin composite modified with different concentrations of Nb/Zn nanoparticles. The investigation of potential biological effects, such as antimicrobial activity, may be addressed in future studies further to explore the multifunctional potential of this nanostructured system.

The statistical null hypothesis tested was that there would be no significant differences in mechanical properties between the unmodified resin composite and the resin composites containing different concentrations of Nb/Zn nanoparticles.

## Material and methods

### Synthesis of zinc-modified niobium nanoparticles and incorporation into the commercial flowable resin composite

To synthesize the hydrated niobium oxide, a 0.26 mol·L⁻¹ solution of the niobium precursor salt, NH₄[NbO(C₂O₄)₂(H₂O)]·3H₂O, was first prepared, followed by the gradual addition of a 1 mol·L⁻¹ NaOH solution under constant mechanical stirring at 300 rpm and a temperature of 65°C. The addition of the alkaline solution stopped when the mixture reached pH 7, indicating the formation of a precipitate, which was then kept in the oven at 70°C for 72 hours for maturation. Then, the precipitate was washed with distilled water using a centrifuge at 7500 rpm for 5 minutes. Finally, the solid was dried in the oven at 70°C for 24 hours, followed by particle size standardization using a 200 mesh sieve. In a subsequent reaction, this material was modified using a solution of zinc nitrate hexahydrate (Zn(NO₃)₂·6H₂O) at 550 ppm of zinc, with the system stirred at 150 rpm for 24 hours at room temperature. Afterward, the supernatant was separated for later analysis, while the resulting solid was washed with distilled water and dried in an oven at 50°C for 24 hours.

Nb/Zn nanoparticles were incorporated into the commercial flowable resin composite OptiFlow II (Nano Flowable Composites, LC/Pac-Dent, Brea, CA, USA) at three different concentrations (0.3%, 0.6%, and 0.9% by weight) for all experimental groups15. The resin composite was weighed using a precision balance with an accuracy of 0.0001g (Denver Instrument, São Paulo, SP, Brazil), and the corresponding amount of nanoparticles was calculated based on the resin's weight percentage. The nanoparticles were weighed and slowly added to the resin, followed by manual homogenization for 1 minute. Four experimental groups were obtained: OptiFlow II (control); OptiFlow II+0.3%Nb/Zn; OptiFlow II+0.6%Nb/Zn; and OptiFlow II+0.9%Nb/Zn.

### Sample size calculation

Sample size calculation was performed using SigmaPlot 12.0 (Systat Software, San Jose, CA, USA), considering a significance level of 5% (α = 0.05) and a statistical power of 80%. The analysis indicated a minimum of 10 specimens per group for evaluating flexural strength and elastic modulus. To account for potential specimen loss during preparation or testing, 14 specimens were initially produced per group. As no specimens were lost, all samples were included in the final analysis. Using a larger sample than the minimum required increased the robustness and reliability of the statistical analysis. 

All specimens were fabricated by a single calibrated operator following standardized preparation procedures to minimize operator-related variability. The experimental groups were previously coded by an independent collaborator who was not involved in specimen preparation, mechanical testing, or data analysis. This coding ensured that the operator responsible for performing the mechanical tests remained blinded to the group allocation during data collection. Statistical analyses were also conducted using the coded dataset, maintaining blinding until the analyses were completed.

### Scanning electron microscopy (sem) and energy-dispersive x-ray spectroscopy (edx)

**Figure 1:**
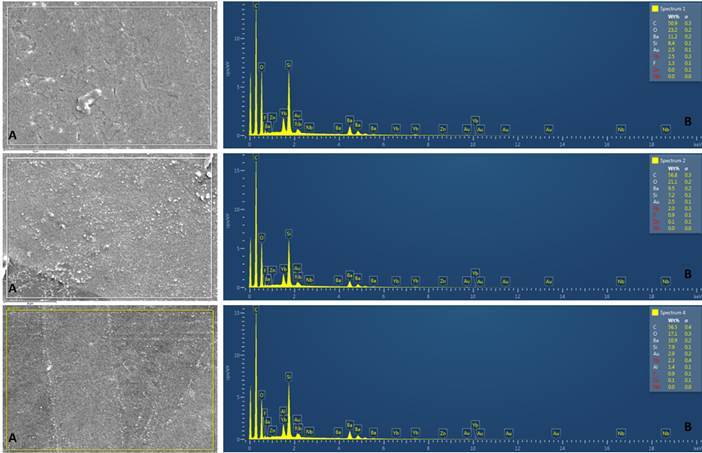
(A) SEM images and (B) their corresponding EDX spectra of OptiFlow II from three different specimens and areas.

**Figure 2:**
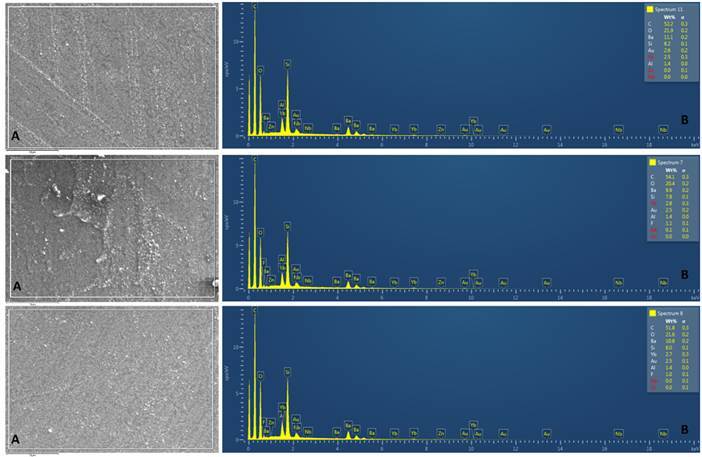
(A) SEM images and (B) their corresponding EDX spectra of OptiFlow II+0.3%Nb/Zn from three different specimens and areas.

**Figure 3:**
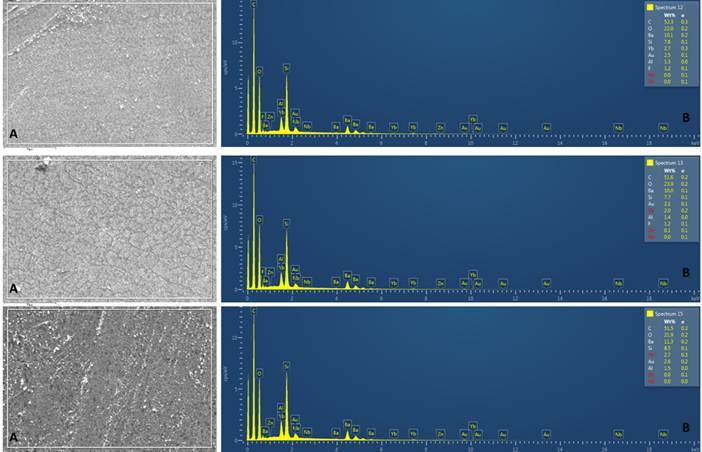
(A) SEM images and (B) their corresponding EDX spectra of OptiFlow II+0.6%Nb/Zn from three different specimens and areas.

**Figure 4:**
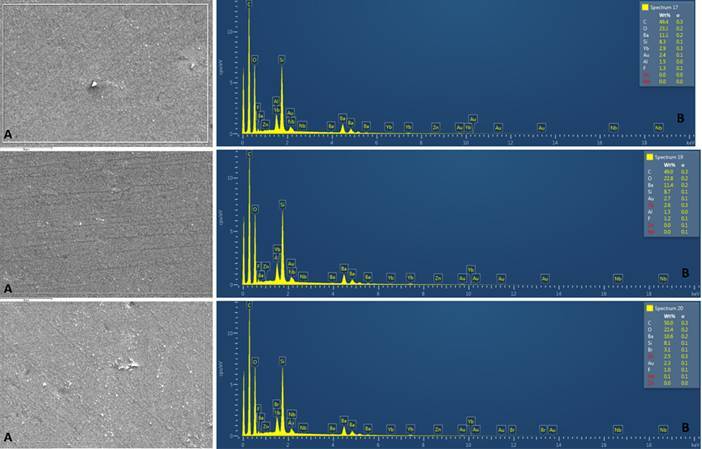
(A) SEM images and (B) their corresponding EDX spectra of OptiFlow II+0.9%Nb/Zn from three different specimens and areas.

For SEM analysis, the samples (n=3) were coated with gold, and the images were acquired at an accelerating voltage of 15 kV with a working distance of 4.5 mm. For EDX analysis (n=3), the X-ray detection system was coupled to a scanning electron microscope (FIB, FEI Helios Nanolab 600i) operating at 20 kV (Figures 1; 2, 3-4).

### Surface michohardness and depth of cure

A metallic mold measuring 2 × 2 mm was used to prepare the specimens (n = 6) ^17^ ^,^ ^18^. The specimens were prepared on a glass plate using a polyester strip, and light-cured through a polyester strip for 40 seconds (LED-Valo Ultradent, 1000 mW/cm²). Subsequently, the specimens were stored in a dry, dark place for 24 hours. Knoop microhardness (KHN) testing was performed by a single operator using a MicroMet 6040 microhardness tester (Buehler LTD, Lake Bluff, IL, USA). According to ISO 4545-1, a diamond indenter with a predetermined load of 50 KgF was applied for 10 seconds. For each sample, three indentations (100 µm apart) were made at the top and bottom surfaces. The mean of the three measurements on each surface was calculated, and the average surface microhardness was determined. To determine the Depth of Cure (DoC), the percentual bottom/top microhardness ratio was calculated. 

### Flexural strength and elastic modulus

Fourteen bars from each group (8×2×2 mm³) were fabricated. The specimens (n=14) were placed in a stainless-steel metallic mold and light-cured for 40 seconds using a 1,000 mW/cm² LED device (LED-Valo Ultradent) ^15^ ^,^ ^17^. The samples were prepared 24 hours prior to testing. Three-point flexural strength was determined using a universal testing machine (Instron, Barueri, SP, Brazil) equipped with a 50 N load cell and a constant crosshead speed of 0.5 mm/min.

The Flexural Strength (FS) and Elastic Modulus (E) values were determined according to equations 1 and 2, respectively:

FS = 3FL/2bh2 1

E = PI3/4bh3d 2

where F is the loading force at the fracture point, I is the distance between the supports (mm), L is the support span length (mm), b is the width, h is the thickness, d is the deflection at load P (mm), and P is the load at a point in the straight-line portion of the load/displacement curve (N).

## Results

### SEM and EDX

Figure 1 represents the OptiFlow II flowable resin composite and its corresponding EDX spectrum. Figures 2A, 3A, and 4A show the distribution of ZnO nanoparticles at concentrations of 0.3%, 0.6%, and 0.9%, respectively, while Figures 2B, 3B, and 4B present their corresponding EDX spectra. The images and spectra were obtained from different areas of the resin composite specimens. It is difficult to visualize the NbZn nanoparticles in SEM images due to their small size and low concentration. However, a homogeneous distribution of nanoparticles within the OptiFlow II flowable resin composite's resin matrix was observed in the groups OptiFlow II+0.3%Nb/Zn, OptiFlow II+0.6%Nb/Zn, and OptiFlow II+0.9%Nb/Zn.

The EDX spectrum showed that Niobium and Zinc were not detectable in the samples, indicating that these low concentrations did not alter the structure of the OptiFlow II flowable resin composite. The spectral lines for Niobium and zinc are shown in all group spectra to highlight their absence, which is likely due to the low concentration of the nanoparticles. The gold peak observed in the spectra corresponds to the metal coating applied to the samples for analysis.

### KHN and DoC

For the statistical analysis of KHN at the top, one-way ANOVA followed by Tukey's test for pairwise comparisons was used. For KHN at the bottom surface, the homoscedasticity assumption was violated; therefore, Welch's ANOVA was used. All analyses were conducted at the α = 5% significance level using R software.

For KHN at the top surface, there was no statistical difference between OptiFlow II, OptiFlow II+0.3%Nb/Zn (p = 0.99), and OptiFlow II+0.9%Nb/Zn (p = 0.99); and between OptiFlow II+0.3%Nb/Zn and OptiFlow II+0.9%Nb/Zn (p = 0.99). There was a significant difference in top KHN between OptiFlow II+0.6% Nb/Zn and OptiFlow II, OptiFlow II+0.3% Nb/Zn, and OptiFlow II+0.9% Nb/Zn (p = 0.008, p = 0.01, and p = 0.007, respectively). The mean and standard deviation (SD) values for the top KHN are presented in Table 1.

**Table 1:** Mean (standard deviation) values for KHN at the top surface. Different letters mean a statistical difference between groups.

	Optiflow II	OptiFlow II+0.3%Nb/Zn	OptiFlow II+0.6%Nb/Zn	OptiFlow II+0.9%Nb/Zn
Mean (SD)	24,3 (2,15)^b^	24,2 (1,8)^b^	20,8 (1,21)^a^	24,3 (1,07)^b^

For KHN at the bottom surface, the data showed a significant difference between OptiFlow II and OptiFlow II+0.3% Nb/Zn (p = 0.04). For all other pairwise comparisons, no statistically significant differences were observed (p > 0.13). The mean and standard deviation (SD) values for the bottom KHN are presented in Table 2.

**Table 2:** Mean (standard deviation) values for KHN at the bottom surface. Different letters mean a statistical difference between groups.

	Optiflow II	OptiFlow II+0.3%Nb/Zn	OptiFlow II+0.6%Nb/Zn	OptiFlow II+0.9%Nb/Zn
Mean (SD)	25,6 (2,79)^b^	21,9 (1,44)^a^	23,5 (4,06)^ab^	22,5 (1,55)^ab^

For DoC, pairwise comparisons were performed using Tukey’s test. The data presented a significant difference between OptiFlow II+0.3%Nb/Zn and OptiFlow II+0.6%Nb/Zn (p = 0.04). For all other pairwise comparisons, no statistically significant differences in DoC values were observed. The following p-values were observed: p = 0.08 for the comparison between OptiFlow II and OptiFlow II+0.3%Nb/Zn; p = 0.88 for OptiFlow II compared to OptiFlow II+0.6%Nb/Zn; p = 0.13 for OptiFlow II compared to OptiFlow II+0.9%Nb/Zn; p = 0.95 for the comparison of OptiFlow II+0.3%Nb/Zn and OptiFlow II+0.9%Nb/Zn; and p = 0.06 for the comparison between OptiFlow II+0.6%Nb/Zn and OptiFlow II+0.9%Nb/Zn. It is noteworthy that the p-value for the last comparison is close to 0.05, indicating that if the significance level were increased to 10%, the null hypothesis of equality would be rejected. Table 3 presents the DoC mean and SD values. 

**Table 3:** Mean (standard deviation) values for DoC. Different letters mean a statistical difference between groups.

	Optiflow II	OptiFlow II+0.3%Nb/Zn	OptiFlow II+0.6%Nb/Zn	OptiFlow II+0.9%Nb/Zn
Mean (SD)	1,06 (0,146)^ab^	0,907 (0,067)^a^	1,13 (0,192)^b^	0,925 (0,059)^ab^

### FS and E

For the statistical analysis of FS, one-way ANOVA followed by Tukey's test for pairwise comparisons was used. For KHN at the bottom surface, the homoscedasticity assumption was violated; therefore, Welch's ANOVA was used. All analyses were conducted at the α = 5% significance level using R software. For E, a generalized linear model (GLM) with a Gamma distribution for the response variable and a log link function was used. Pairwise comparisons were performed using Tukey's test with a sandwich-type covariance matrix. All analyses were performed at the α = 5% significance level using R software.

The FS data did not show significant differences between OptiFlow II+0.3% Nb/Zn and OptiFlow II+0.6% Nb/Zn (p = 0.95), or between OptiFlow II+0.3% Nb/Zn and OptiFlow II+0.9% Nb/Zn (p = 0.10). Similarly, no statistically significant difference was observed between OptiFlow II+0.6% Nb/Zn and OptiFlow II+0.9% Nb/Zn (p = 0.29). However, there were significant differences between OptiFlow II and all experimental groups, with p-values of 0.02, 0.003, and <0.0001 for comparisons with OptiFlow II+0.3% Nb/Zn, OptiFlow II+0.6% Nb/Zn, and OptiFlow II+0.9% Nb/Zn, respectively. The mean and standard deviation (SD) values for FS are presented in Table 4.

**Table 4:** Mean (standard deviation) values for FS. Different letters mean a statistical difference between groups.

	Optiflow II	OptiFlow II+0.3%Nb/Zn	OptiFlow II+0.6%Nb/Zn	OptiFlow II+0.9%Nb/Zn
Mean (SD)	80,1 (7,36)^b^	72,5 (6,92)^a^	71,2 (5,91)^a^	66,8 (5,21)^a^

Regarding E, the data did not show a significant difference between OptiFlow II and OptiFlow II+0.3%Nb/Zn (p = 0.72), nor between OptiFlow II+0.6%Nb/Zn and OptiFlow II+0.9%Nb/Zn (p = 0.98). A borderline p-value was observed for the comparison between OptiFlow II+0.3%Nb/Zn and OptiFlow II+0.9%Nb/Zn (p = 0.06). However, considering a higher significance level, such as 10%, the null hypothesis of equality in E between these two groups would be rejected. Statistical difference was observed between OptiFlow II and OptiFlow II+0.6%Nb/Zn (p = 0.01); OptiFlow II and OptiFlow II+0.9%Nb/Zn (p = 0.02), and OptiFlow II+0.3%Nb/Zn and OptiFlow II+0.6%Nb/Zn (p = 0.02). The mean and SD values of E are presented in Table 5. 

**Table 5:** Mean (standard deviation) values for E. Different letters mean statistical difference between groups.

	Optiflow II	OptiFlow II+0.3%Nb/Zn	OptiFlow II+0.6%Nb/Zn	OptiFlow II+0.9%Nb/Zn
Mean (SD)	3.56 (0,27)^c^	3.48 (0,15)^bc^	3.3 (0,18)^a^	3.3 (0,27)^ab^

## Discussion

Failures of resin composite restorations are mainly associated with fractures or caries adjacent to materials; therefore, improving the mechanical and antibacterial properties of these materials may enhance their clinical performance and increase the longevity of restorations. ^5^ ^,^ ^10^ ^,^ ^11^ ^,^ ^12^ ^,^ ^19^ ^,^ ^20^ ^,^ ^21^ ^,^ ^25^ ^,^ ^26^ ^,^ ^27^. In this context, previous studies have shown that the addition of ZnO and Nb nanoparticles seems promising for enhancing the antibacterial activity of resin composites without compromising their mechanical properties ^6^ ^,^ ^10^ ^,^ ^11^ ^,^ ^12^ ^,^ ^13^ ^,^ ^14^ ^,^ ^15^ ^,^ ^28^ ^,^ ^29^. However, incorporating nanoparticles into resin-based materials may also introduce structural challenges, particularly regarding particle dispersion, interfacial interactions with the polymer matrix, and potential effects on mechanical performance. Therefore, the present study sought to investigate whether incorporating zinc-modified niobium nanoparticles into a flowable resin composite would influence its mechanical behavior.

The results of the present study partially agree with these previous findings, as the depth of cure of the tested flowable resin composite was not affected by the incorporation of NbZn nanoparticles; however, FS was significantly reduced, regardless of the nanoparticle concentration. The reduction in FS observed in the experimental groups with NbZn addition compared to the control group may be attributed to the fact that ZnO is an opaque particle, which can interfere with light transmission ^20^ ^,^ ^22^, and that the absence of a silane treatment of the Nb/Zn nanoparticles might have caused a weak adhesion of the nanoparticles to the resin matrix ^19^. Nevertheless, it is important to note that the absence of silanization does not necessarily imply the absence of interaction between the inorganic particles and the organic matrix. Previous studies have demonstrated that, although silane coupling agents are commonly used to promote chemical bonding between inorganic fillers and the organic resin matrix, unsilanized nanoparticles may still interact physically with the polymer network. These interactions may occur through mechanisms such as van der Waals forces, mechanical interlocking, and interfacial friction, thereby contributing to partial stress transfer within the composite structure. In addition, the presence of non-chemically bonded filler-matrix interfaces may allow a certain degree of relative movement between the inorganic particles and the polymer matrix, influencing stress distribution and energy dissipation within the material. Consequently, although the absence of silanization may reduce stress-transfer efficiency compared with chemically bonded fillers, unsilanized nanoparticles can still affect the mechanical behavior of resin composites through physical interfacial interactions ^30^ ^,^ ^31^.

The mechanical properties of restorative materials are important indicators of their clinical applicability, as satisfactory mechanical performance reduces the likelihood of material fractures ^15^ ^,^ ^22^ ^,^ ^25^. Several experimental tests are commonly used to evaluate the mechanical behavior of resin composites, including microhardness, depth of cure, flexural strength, and elastic modulus. These properties were evaluated in the present study. Regarding KHN, the results of the present study are in agreement with previous studies ^6^ ^,^ ^19^ ^,^ ^22^ that evaluated the incorporation of Zinc and Niobium, either individually or as nanohybrids combined with other agents, and also found that lower nanoparticle concentrations did not affect the microhardness values of the resin composite, showing results similar to the control group. 

Regarding the DoC property, as observed in the present study, previous studies ^12^ ^,^ ^15^ ^,^ ^19^ ^,^ ^20^ ^,^ ^21^ that incorporated Niobium or zinc into resin composites have shown that, at low concentrations, these agents do not affect the depth of cure of the materials. In general, the depth of cure tends to decrease as the inorganic particle content increases, due to light scattering at the resin-filler interfaces ^15^ ^,^ ^20^ ^,^ ^21^ ^,^ ^22^ and the opacity of ZnO nanoparticles to visible light, which may also negatively influence the polymerization of the resins ^20^. However, despite the increase in NbZn nanoparticle concentration, the present study did not observe a reduction in the DoC. This result may be attributed to the low overall nanoparticle content and to the use of a hybrid compound combining Zn with Nb, as Niobium is known to exhibit photocatalytic activity, which may have enhanced the polymerization process, thereby preventing a decrease in DoC ^15^ ^,^ ^22^.

Although the KHN and DoC of the flowable resin composite in the present study were not affected by the incorporation of low concentrations of NbZn nanoparticles, FS was significantly reduced at all tested concentrations. In the study by Wang et al. ^22^, only the group with the lowest ZnO concentration showed FS comparable to that of the control group. In contrast, all other concentrations reduced this property. Lee et al. ^19^ also reported a reduction in the FS of flowable resin composites after the addition of a zinc-based hybrid compound. Similarly to the present study, the particles added were not silanized, which may explain the decrease in FS observed in the experimental groups, as the absence of silane treatment can compromise the homogenization of nanoparticles within the resin matrix, leading to agglomeration and, consequently, reducing the mechanical properties of the material ^15^ ^,^ ^19^ ^,^ ^22^. From a materials design perspective, this finding should not be interpreted solely as a justification for the reduction in flexural strength, but rather as an indication that further optimization of nanoparticle surface treatment or dispersion strategies may be necessary to maximize the reinforcing potential of these nanostructures.

In the present study, although adequate distribution of Nb/Zn nanoparticles was observed in the tested fluid resin, as evidenced by the preservation of the structure in SEM images and EDX, a reduction in flexural strength was observed for all groups with the addition of NbZn nanoparticles. Therefore, although the modification did not improve FS under the conditions tested, the results contribute to the understanding of how nanoparticle incorporation influences the mechanical behavior of flowable resin composites and may guide future material optimization strategies. Although the FS values of the groups containing NbZn nanoparticles were lower than those of the control group, they remained above the minimum acceptable value established by ISO 4049:2009 (International Organization for Standardization Technical Committee, 2009), which states that the minimum flexural strength required for flowable resin composites is 50 MPa ^6^. However, meeting the ISO minimum requirement does not necessarily indicate superior clinical performance; rather, it confirms that the material remains within the acceptable mechanical range defined for this class of restorative materials. Therefore, these findings should be interpreted cautiously, particularly when considering potential clinical applications.

Regarding E, the group with the lowest nanoparticle addition in the present study (OptiFlow II+0.3% Nb/Zn) exhibited an elastic modulus comparable to that of the control group. This finding agrees with previous studies, which also reported no significant changes in the elastic modulus following the incorporation of Niobium or Zinc nanoparticles ^15^ ^,^ ^19^ ^,^ ^20^ ^,^ ^22^. Some limitations of this study should also be acknowledged. In an in vitro investigation, the experimental conditions cannot fully replicate the complex mechanical, chemical, and biological challenges of the oral environment. In addition, only mechanical properties were evaluated, whereas other relevant characteristics, such as long-term degradation, wear resistance, and antibacterial performance, were not assessed. Furthermore, the nanoparticles used in this study were not surface-treated with silane, which may have influenced their interactions with the polymer matrix and, consequently, affected mechanical outcomes. Future studies should investigate alternative surface functionalization strategies, as well as the long-term physicochemical stability and biological properties of these materials.

Thus, the present study demonstrated that incorporating zinc-modified niobium nanoparticles at varying concentrations into the OptiFlow II flowable resin composite influences its mechanical properties. Lower concentrations showed performance comparable to the control group, whereas higher concentrations negatively affected these properties, highlighting the importance of optimizing nanoparticle loading. These findings provide valuable insights into the influence of Nb/Zn nanostructures on the mechanics of resin composites and may support the development of future multifunctional restorative materials.

## Data Availability

The research data are available upon request.
